# The Outcome of Immediate Adjuvant Postoperative External Beam Radiotherapy Versus Observation Alone After Radical Prostatectomy in High-Risk Prostate Cancer: A Meta Analysis

**DOI:** 10.3390/jcm15083149

**Published:** 2026-04-20

**Authors:** Walaa Borhan, Emad Rajih

**Affiliations:** 1 Pathology Unit, Basic Medical Science Department, College of Medicine, Taibah University, Madinah 42353, Saudi Arabia; wborhan@taibahu.edu.sa; 2 Urology Unit, General and Specialized Surgery Department, College of Medicine, Taibah University, Madinah 42353, Saudi Arabia

**Keywords:** prostate cancer, adjuvant radiotherapy, radical prostatectomy, high-risk, metastasis-free survival, progression-free survival, meta-analysis

## Abstract

**Background**/**Objectives**: High-risk prostate cancer patients undergoing radical prostatectomy remain at significant risk of biochemical recurrence and metastasis. Immediate adjuvant external beam radiotherapy (EBRT) has been proposed to improve outcomes, but its role compared to observation remains debated due to potential toxicity and uncertain overall survival benefit. To evaluate the efficacy and safety of immediate adjuvant EBRT versus observation following radical prostatectomy in men with high-risk prostate cancer. **Methods**: A meta-analysis was conducted according to PRISMA guidelines. We sought to include both randomized controlled trials (RCTs) and observational studies published between 2005 and 2025; however, no observational studies meeting the predefined criteria were identified. Therefore, only RCTs comparing immediate adjuvant EBRT with observation in patients with adverse pathological features and undetectable postoperative PSA were included. Primary outcomes were biochemical recurrence-free survival (BCR-FS), metastasis-free survival (MFS), and overall survival (OS). Secondary outcomes included toxicity and quality of life (QoL). Data were pooled using Mantel–Haenszel and inverse variance methods, and heterogeneity was assessed with I^2^ statistics. **Results**: Four RCTs (n = 1987) met the inclusion criteria. Adjuvant EBRT significantly improved progression-free survival (PFS) (HR = 0.38; 95% CI: 0.20–0.74; *p* = 0.004) and metastasis-free survival (HR = 0.70; 95% CI: 0.54–0.92; *p* = 0.01). However, OS benefit was not statistically significant (HR = 0.88; 95% CI: 0.59–1.32; *p* = 0.55). Heterogeneity was substantial for some outcomes (I^2^ up to 71%). Adjuvant EBRT was associated with higher genitourinary and gastrointestinal toxicity compared to observation. **Conclusions**: Immediate adjuvant EBRT after radical prostatectomy improves PFS and MFS in high-risk prostate cancer but does not confer a clear OS advantage. Treatment decisions should be individualized, balancing disease-control benefits against toxicity risks. Observation with early salvage RT remains a reasonable alternative in selected patients.

## 1. Introduction

Prostate cancer is the second most common neoplasm in men after lung cancer and accounts for approximately 1,276,106 new cases and 358,989 deaths worldwide in 2018, corresponding to approximately 3.8% of all male cancer death [1,2]. Prevalence and mortality are directly associated with advancing age, with an average of 66 years at diagnosis [3,4].

Prostatic cancer is typically asymptomatic in its initial phase and characteristically evolves slowly, often requiring minimal or no emergency treatment [5,6]. When symptoms do arise, they are most commonly urinary difficulty, frequency, and nocturia that also may overlap symptoms associated with benign prostate hyperplasia [7]. Later in the course, patients may develop urinary retention and skeletal pain, especially the axial skeleton since it is the most frequent location for metastatic bone cancer [4,8].

Although detection and monitoring are eased through the application of prostate-specific antigen (PSA) screening [9], PSA testing is not the specific biomarker for cancer because elevated PSA is also an indicator of benign prostatic hyperplasia or other non-malignant pathology [10,11]. The prostate-specific antigen density (PSAD), expressed in terms of prostate volume (PV) is now a better predictor of prostate cancer as a diagnostic improvement [12,13]. While PV is estimable by digital rectal examination or transrectal ultrasound (TRUS) [14], these methods have limitations in accuracy and inter-operator variability [15,16]. In contrast, magnetic resonance imaging (MRI) is more precise to estimate PV and consequently facilitates PSAD calculation during the time of biopsy for improved risk stratification [17,18].

Adjuvant radiotherapy has been described as radiotherapy (RT) administration after radical prostatectomy (RP) in those with adverse pathological characteristics and an undetectable PSA level to limit the threat of recurrence [19]. While RP is a gold-standard curative therapy in localized prostate cancer [20], patients with high-risk features such as positive surgical margin, extracapsular extension, or seminal vesicle invasion are nevertheless at significant risk of biochemical recurrence (BCR), distant metastasis, and cancer-specific death [21]. In these patients, the application of early postoperative external beam radiotherapy (EBRT) has been studied extensively as a method to eradicate microscopic residual disease and improve long-term outcomes [22,23].

Early adjuvant EBRT in principle reduces BCR by curing left-over cancer cells, which is supported by randomized controlled trials (RCTs) such as SWOG 8794 [19], EORTC 22911, and ARO 96-02 [24]; however, it is accompanied by urinary and bowel toxicities that may raise issues of overtreatment as well as quality of life (QoL) [25,26]. This has led to continued debate regarding whether observation with early salvage radiotherapy (sRT) at relapse might be a superior alternative for carefully selected patients as demonstrated in the trials such as RADICALS-RT [19,27,28,29]. A prospective systematic review and meta-analysis study [30] compared modern RCTs such as RADICALS-RT, RAVES, and GETUG-AFU-17; however, these trials did not include strict adjuvant-versus-observation arms and therefore address a different clinical question. While there are published systematic reviews and meta-analyses [31,32] that provide essential insights into the benefits of adjuvant EBRT, they often include limited QoL data and lack a strict focus on immediate adjuvant therapy in clearly defined high-risk pathological subgroups. Given these uncertainties, it is important to synthesize existing evidence to determine if immediate postoperative adjuvant EBRT is associated with a substantial survival advantage that justifies its potential harms. Although our protocol permitted the inclusion of observational studies, no such studies met the predefined eligibility criteria; therefore, this review updates Morgan et al. (2008) [31] by extending the search through 2025 and synthesizing randomized controlled trial evidence comparing immediate adjuvant EBRT with observation in high-risk men following radical prostatectomy, with the aim of informing clinical practice and guideline development.

## 2. Methodology

### 2.1. Study Design

This meta-analysis was conducted in accordance with the Preferred Reporting Items for Systematic Reviews and Meta-Analyses (PRISMA) guidelines [33]. We were unable to complete PROSPERO registration because data extraction and analysis had already begun, rendering the review ineligible for registration under PROSPERO’s requirements. The objective was to compare immediate adjuvant external beam radiotherapy (EBRT) with observation following radical prostatectomy in patients with high-risk prostate cancer.

### 2.2. Eligibility Criteria

Eligible studies included randomized controlled trials (RCTs), prospective and retrospective cohort studies, and comparative observational studies published in English from 2005 to 2025. The period of inclusion was widened to cover all the best-quality evidence that would reflect the change in surgical procedures, RT protocols, and risk stratification strategies over the last two decades to allow for a thorough and representative integration of data.

The target population included adult men (≥18 years) who received radical prostatectomy and were high-risk according to positive surgical margins, extracapsular extension, seminal vesicle invasion, or T3 stage disease but had undetectable PSA after surgery and no lymph node involvement. The intervention was early adjuvant EBRT that was delivered within six months after surgery with no preceding biochemical recurrence and no concurrent androgen deprivation therapy (ADT). The control was observation with no immediate postoperative treatment.

Primary endpoints were BCR-FS, overall survival (OS), and metastasis-free survival (MFS). Secondary endpoints were treatment-related toxicity and QoL.

### 2.3. Search Strategy

A comprehensive literature search was performed in PubMed, EBSCOhost databases, and through manual reference screening. The PubMed search strategy combined MeSH terms and keywords using Boolean operators:

(“prostatic neoplasms*” OR “prostate cancer*” OR “prostate neoplasm*”) AND (“prostatectomy*” OR “radical prostatectomy*”) AND (“adjuvant radiotherapy*” OR “external beam radiotherapy*” OR “EBRT” OR “postoperative radiotherapy*”) AND (“observation*” OR “watchful waiting*” OR “active surveillance*” OR “monitoring*” OR “Wait-and-See”) AND (“high risk*” OR “gleason score*” OR “positive surgical margin*” OR “extracapsular extension*” OR “seminal vesicle invasion*” OR “t3 stage*”) AND (“survival*” OR “prognosis*” OR “Metastasis-Free Survival”)

Filters applied: English language, publication date from 1 January 2005 to 2025. Similar strategies were adapted for EBSCOhost. Manual searches identified additional relevant studies.

### 2.4. Study Selection and Data Extraction

All retrieved records were imported into reference management software, and duplicates were removed. Two independent reviewers screened titles and abstracts, followed by full-text review. Discrepancies were resolved by consensus or a third reviewer. In addition to database searching, we performed manual reference screening of all included articles and relevant reviews to identify any additional eligible studies. No automation tools were used for screening or eligibility assessment. These steps follow PRISMA 2020 recommendations and ensure transparency and reproducibility in the study selection process. Data extraction was performed using a standardized form, capturing study characteristics, patient demographics, intervention details, comparators, and outcomes (BCR-FS, OS, MFS, toxicity, QoL). Effect measures (hazard ratios, odds ratios, risk ratios) with 95% confidence intervals were recorded. The Cochrane risk of bias tool [34] was used for the studies.

### 2.5. Statistical Analysis

Meta-analysis was conducted using RevMan 5.3 [35] (Cochrane Collaboration, Copenhagen, Denmark). Hazard ratios (HR) and odds ratios (OR) were pooled using the inverse variance and Mantel–Haenszel methods with 95% confidence intervals (CIs). A fixed-effects model was applied when heterogeneity was low (I^2^ < 50%), and a random-effects model was applied when heterogeneity was substantial (I^2^ > 50%). Heterogeneity was assessed using Cochran’s Q and I^2^ statistics. Publication bias was evaluated using funnel plots. A *p*-value < 0.05 was considered statistically significant.

### 2.6. Risk of Bias and Quality Assessment

The Cochrane risk of bias tool was used to assess risk of bias, and study quality was considered when interpreting findings [36]. The GRADE certainty of evidence was determined for the eligible studies based on the outcome and categorized as high, moderate, low, or very low depending on study quality [37].

## 3. Result

### 3.1. Study Selection

The initial database search yielded 76 records, of which 5 duplicates were removed. After screening titles and abstracts, 26 full-text articles were assessed for eligibility. Ultimately, four randomized controlled trials (RCTs) met the inclusion criteria and were included in the qualitative and quantitative synthesis. These were SWOG S8794 [38], EORTC 22911 [39], ARO 96-02 [40], and NCT02668718 [41].

No observational studies meeting the predefined inclusion criteria were identified. Several large-scale retrospective analyses compared radical prostatectomy with EBRT as primary treatment modalities; however, these studies did not evaluate adjuvant EBRT following radical prostatectomy versus observation and were therefore excluded. Therefore, the quantitative synthesis presented here is based exclusively on four randomized controlled trials.

**Figure 1:**
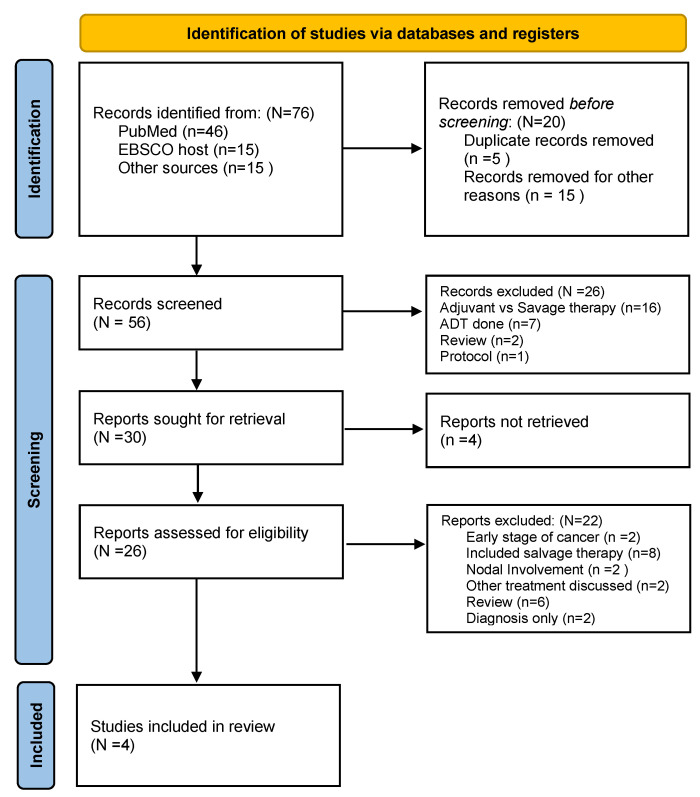
PRISMA flow diagram.

A PRISMA flow diagram summarizing the study selection process is presented in (Figure 1).

### 3.2. Characteristics of Included Studies

Four major RCTs collectively enrolled 1987 patients with high-risk prostate cancer who had undergone radical prostatectomy. The trials were conducted across multiple countries, including the USA, Europe, Germany, and Finland, with median follow-up durations ranging from 9.3 to 12.6 years, ensuring robust long-term outcome data.

Eligibility criteria across trials were broadly similar, focusing on patients with adverse pathological features such as extracapsular extension (ECE), positive surgical margins, or pT3 disease. While SWOG S8794 [38] and EORTC 22911 [39] included patients with seminal vesicle invasion, NCT02668718 [41] excluded these cases, limiting its population to pT2 with positive margins or pT3a disease. All trials required undetectable or low postoperative PSA levels prior to randomization.

Adjuvant EBRT protocols varied slightly among studies. SWOG S8794 [38] and EORTC 22911 [39] delivered 60–64 Gy using two-dimensional techniques, whereas ARO 96-02 [40] and NCT02668718 [41] employed three-dimensional conformal RT, with NCT02668718 [41] using a higher dose of 66.6 Gy. The timing of adjuvant EBRT ranged from 6 to 18 weeks post-surgery. Observation arms in all trials allowed sRT upon biochemical or clinical progression, with approximately one-third of patients in the observation groups eventually receiving salvage treatment.

Primary endpoints differed across studies: SWOG S8794 [38] focused on MFS, EORTC 22911 [39] and NCT02668718 [41] evaluated biochemical progression-free survival (PFS), and ARO 96-02 [40] assessed PFS. All trials reported treatment-related toxicity, primarily genitourinary and gastrointestinal adverse effects, with higher rates observed in the adjuvant EBRT arms.

A summary of key protocol characteristics is presented in Table 1.

**Figure 2:**
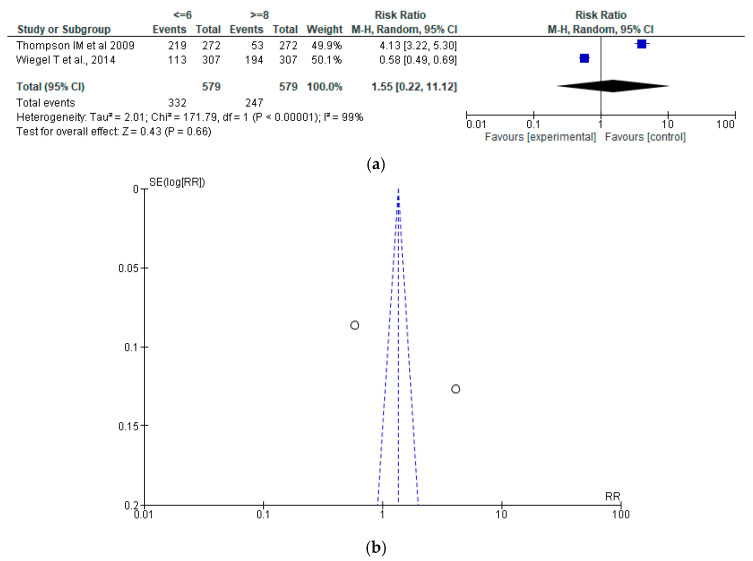
(**a**) Forest plot for Gleason score [38,40]; (**b**) Funnel plot for Gleason Score.

Gleason score (exploratory analysis):

The pooled analysis showed no significant difference between groups (RR = 1.55, 95% CI: 0.22–11.12; *p* = 0.66). Interpretation is limited by extreme heterogeneity (I^2^ = 99%) and the small number of studies with conflicting results. Therefore, these findings should be considered exploratory rather than conclusive (Figure 2a,b).

### 3.3. Metastasis-Free Survival

**Figure 3:**
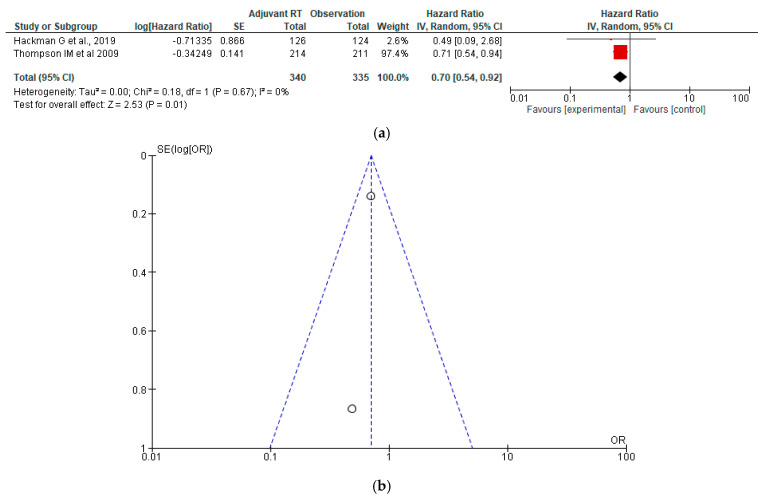
(**a**) Forest plot for MFS between adjuvant EBRT and observation [38,41]; (**b**) Funnel plot for MFS between adjuvant EBRT and observation.

The forest plot presents a meta-analysis comparing adjuvant EBRT with observation, using Hazard ratios (HRs) as the effect measure [38,41]. The pooled hazard ratio is 0.70 [95% CI: 0.54 to 0.92], suggesting that patients receiving adjuvant EBRT had 30% lower odds of experiencing the outcome compared to those under observation. This result is statistically significant (Z = 2.53, *p* = 0.01) and favours the experimental (RT) group. The two studies included showed consistent results, and heterogeneity is negligible (I^2^ = 0%, Chi^2^ = 0.18, *p* = 0.67), indicating strong agreement between the studies. The random-effects model accounts for potential differences in study design, though here, between-study variation is minimal (Figure 3a,b).

### 3.4. Overall Survival

**Figure 4:**
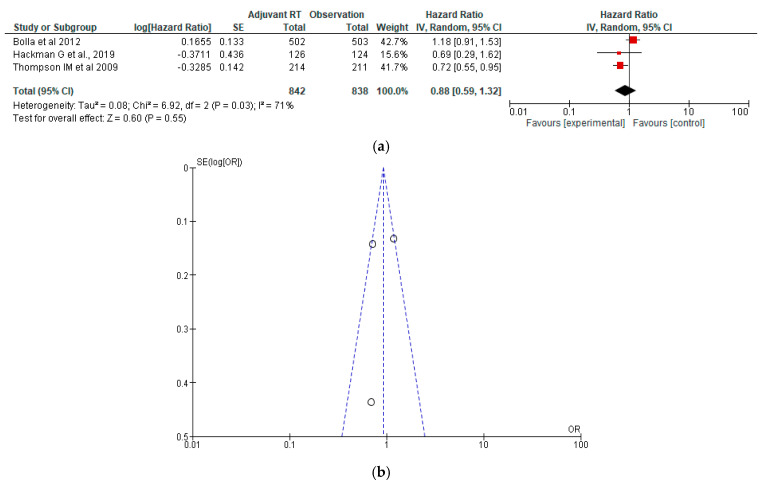
(**a**) Forest plot for overall survival between adjuvant EBRT and observation [38,39,41]; (**b**) Funnel plot for overall survival (OS) between adjuvant EBRT and observation.

The forest plot shows a meta-analysis of three studies comparing adjuvant EBRT versus observation using Hazard ratios (HRs). The pooled hazard ratio is 0.88 [95% CI: 0.59 to 1.32], indicating a non-significant 12% reduction in odds of the outcome in the adjuvant EBRT group compared to observation. The test for overall effect is not statistically significant (Z = 0.60, *p* = 0.55), meaning no clear benefit of adjuvant EBRT was observed across these studies. Heterogeneity is substantial (I^2^ = 71%, Chi^2^ = 6.92, *p* = 0.03), suggesting notable variation in effect sizes between studies. While one study [38] showed a significant benefit, others did not, and the overall findings remain inconclusive due to inconsistency and lack of statistical significance (Figure 4a,b).

### 3.5. Progression-Free Survival (PFS)

**Figure 5:**
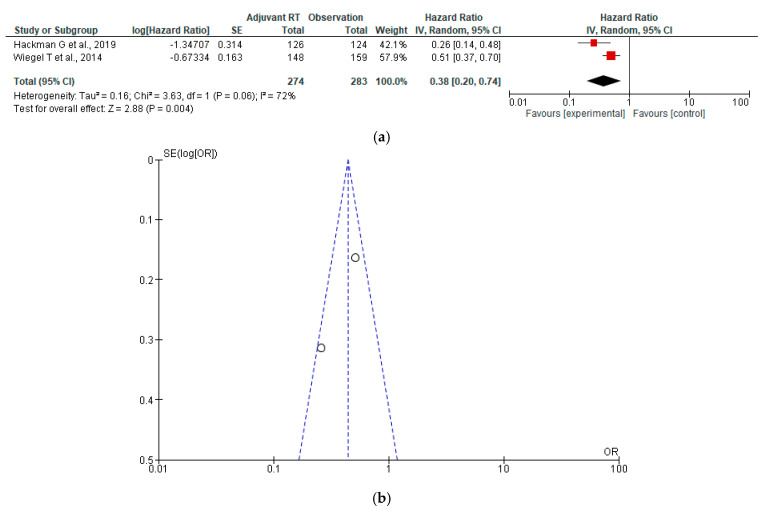
(**a**) Forest plot for progression-free survival (PFS) between adjuvant EBRT and observation [40,41]; (**b**) Funnel plot for progression-free survival (PFS) between adjuvant EBRT and observation.

The forest plot presents a meta-analysis of two studies comparing adjuvant EBRT with observation, using hazard ratios (HRs) to assess outcomes [40,41]. The pooled hazard ratio is 0.38 [95% CI: 0.20 to 0.74], indicating that patients receiving adjuvant EBRT had 62% lower odds of the adverse outcome compared to those under observation a statistically significant difference (Z = 2.88, *p* = 0.004). Both studies individually favoured the RT group, with confidence intervals not crossing 1. However, heterogeneity is substantial (I^2^ = 72%, Chi^2^ = 3.63, *p* = 0.06), suggesting variability in effect size across studies. Despite this, the overall result supports the benefit of adjuvant EBRT in reducing the targeted adverse outcome (Figure 5a,b).

### 3.6. Toxicity and Quality of Life

The four included randomized trials reported toxicity and QoL outcomes using different grading systems and assessment tools, leading to heterogeneity that prevented quantitative pooling. However, when synthesized narratively, consistent patterns were evident. Genitourinary and gastrointestinal toxicities were uniformly higher in the adjuvant EBRT arms across studies, particularly for Grade 1–2 urinary frequency, urgency, and bowel irritation, as reported in SWOG 8794 and ARO 96-02 [38,40]. Higher-grade events (Grade ≥ 3) were noted predominantly in EORTC 22911 and NCT02668718, with the latter reporting substantially increased rates of severe adverse events, including urethral strictures requiring intervention [39,41]. Early (0–6 months) bowel and urinary symptoms were more pronounced with RT, consistent with SWOG 8794, while late effects (≥2–3 years), particularly urethral strictures and persistent urinary dysfunction, were more frequently documented in the Finnish trial [38] (Table 2).

QoL findings also varied across trials due to the use of different validated tools, including EPIC and SF-12 (RAVES), IIEF-5, LENT-SOMA, and IPSS (NCT02668718), and study-specific questionnaires (SWOG 8794) [38,41]. When stratified by instrument, early QoL deterioration was consistently observed in bowel and urinary domains following adjuvant EBRT, whereas erectile dysfunction showed high baseline prevalence in both arms and minimal long-term divergence. LENT-SOMA analyses in NCT02668718 demonstrated significantly higher intestinal toxicity scores in the RT arm at late follow-up, while IPSS scores suggested a trend toward worsened urinary symptoms during intermediate follow-up [41]. By contrast, SWOG 8794 showed recovery of global QoL by two years post-treatment, indicating partial resolution of acute RT-related effects [38]. Due to differences in instruments and timing, pooled analysis was not feasible, but the structured stratified summary provides a clearer, integrated understanding of toxicity and QoL patterns across trials (Table 2).

### 3.7. Risk of Bias

The risk of bias across the four included RCTs was generally rated as presenting “some concerns,” primarily due to their open-label design, which affects subjective outcomes such as patient-reported quality-of-life measures and clinician-graded toxicity. Randomization processes, allocation concealment, and completeness of follow-up were adequately documented in all trials, resulting in low risk for these domains, and objective outcomes such as biochemical recurrence, metastasis, and survival were judged at low risk of measurement bias. Overall, the absence of blinding and incomplete transparency in prespecified analyses contributed to an “overall some concerns” judgement across all trials (Table S1).

### 3.8. GRADE Certainty of Assessment

The certainty of evidence was assessed using the GRADE approach, applying conservative judgments appropriate for a dataset consisting of only four RCTs. Progression-free survival was rated as moderate certainty due to substantial heterogeneity despite a consistent direction of benefit, while metastasis-free survival was also rated moderate because the effect favoured adjuvant EBRT but was based on only two trials with confidence intervals close to the null. Overall survival was downgraded to low certainty owing to both inconsistency and wide confidence intervals crossing unity. Serious toxicity was assessed as moderate certainty, with consistent evidence of higher grade ≥3 genitourinary events but some risk of bias related to open-label designs and subjective grading. Quality-of-life evidence was rated low certainty because of unblinded patient-reported outcomes, variation in measurement tools, and incomplete reporting across studies. Reporting bias could not be formally assessed because fewer than ten studies contributed to each outcome, making publication-bias testing unreliable under Cochrane guidance; therefore, reporting bias was appropriately classified as “Not Applicable” (Table S2).

## 4. Discussion

### 4.1. Principal Findings

The final evidence base comprised only randomized controlled trials, ensuring that the conclusions of this review reflect high-level comparative data. This RCT-only framework strengthens the validity of the pooled survival outcomes and aligns the interpretation strictly with trial-based evidence that compared immediate adjuvant postoperative EBRT with observation after radical prostatectomy in patients at a high risk of prostate cancer. The findings reveal that adjuvant EBRT enhances significantly both PFS and MFS, lowering the probability of progression by 62% and metastasis by 30%, respectively. These results are consistent with earlier randomized trials that have proved early RT postpones recurrence of disease and metastatic dissemination [38,40]. Yet, OS was not significantly different in a statistical manner, and substantial heterogeneity (I^2^ = 71%) indicates variation in patient choice, follow-up, and treatment techniques among the studies.

### 4.2. Comparison with Previous Study

Our review updates the prior systematic review by Morgan et al. (2008) [31] by extending the search period through 2025 and by including an additional randomized trial NCT02668718 [41] not available in the earlier synthesis. Unlike the 2008 analysis, which pooled three RCTs and reported strong biochemical control but no OS, our pooled dataset (four RCTs, n ≈ 1987) demonstrates significant progression-free survival and MFS with immediate adjuvant EBRT, while OS remains non-significant. We also applied contemporary review standards (PRISMA) and reported updated RT techniques and doses (including 3D conformal RT and higher dose in the NCT02668718 [41]). These additions provide a more current assessment of adjuvant EBRT in the era of modern RT and facilitate discussion of current strategies (immediate adjuvant vs. early salvage) considering contemporary trials and practice.

Importantly, the present analysis is intentionally restricted to older adjuvant-RT-versus-observation randomized trials, as these trials directly address the clinical question under investigation. Modern trials evaluating adjuvant versus early sRT (RADICALS-RT, RAVES, GETUG-AFU-17) examine a fundamentally different therapeutic comparison and therefore did not contribute data to this evidence synthesis. Their role is acknowledged solely for contextual clarity, while the analytic conclusions remain grounded exclusively in the RCTs that compared immediate adjuvant EBRT with observation.

### 4.3. Clinical Implications

The increase in progression-free survival and MFS support the use of adjuvant EBRT for patients with unfavourable pathological parameters of positive surgical margins, extracapsular extension, or seminal vesicle invasion [32,42,43,44]. These benefits are clinically relevant because postponing recurrence and metastasis potentially reduces the need for systemic treatment and its resultant morbidity [45,46]. However, the absence of a clear OS advantage and the potential for treatment-related toxicity—such as urinary incontinence, erectile dysfunction, and bowel complications—necessitate individualized decision-making [47,48,49]. Clinician’s consideration on the patient’s age, comorbidities, and life expectancy is advisable in the decision to recommend adjuvant EBRT [50,51]. Shared decision-making is essential so that the patients understand both the attainable benefit in the control of the disease and the risk of functional impairment [49,52]. In established healthcare settings with strict PSA monitoring and expedited sRT availability, observation with early salvage therapy can still be an acceptable choice, particularly for those patients who value QoL [53,54].

## 5. Strengths and Limitations

This review has several strengths. It addresses a clinically important question using a clearly defined population of high-risk prostate cancer patients with adverse pathological features after radical prostatectomy. The analysis is based exclusively on randomized controlled trials, enhancing the reliability of the findings. Our use of a standardized Cochrane methodology, PRISMA-guided reporting, and the synthesis of multiple meaningful endpoints, including progression-free survival, metastasis-free survival, overall survival, toxicity, and quality of life, contribute to the robustness of the conclusions.

However, certain limitations must be acknowledged. The number of eligible RCTs was limited, and heterogeneity was observed for some outcomes. Reliance on aggregate rather than individual patient data restricted detailed subgroup analyses. Variability in radiotherapy techniques, follow-up protocols, the reporting of toxicity, and quality-of-life instruments across trials also limits comparability. Finally, differences between historical RT techniques in the included trials and modern RT practice may affect generalizability to current clinical settings.

## 6. Future Directions

Large-scale randomized controlled trials, where adjuvant EBRT is compared with early sRT, are the priority for future research to define the ideal timing of postoperative RT. The integration of molecular and genomic classifiers into risk stratification models may assist in the identification of patients most likely to gain benefit from immediate RT. Moreover, investigations must assess long-term QoL, toxicity patterns, and cost utility to inform evidence-based, practice-based decision-making. RT technology improvements, including image-guided and intensity-modulated RT, must also be investigated to optimize the reduction in treatment morbidity with preserved oncological efficacy.

## 7. Conclusions

Adjuvant EBRT following radical prostatectomy in high-risk prostate cancer considerably enhances progression-free and MFS but does not show a definitive OS benefit. These results justify selective utilization of adjuvant EBRT in patients with unfavourable pathological characteristics based on individualized risk stratification and patient preferences. Ongoing studies evaluating adjuvant EBRT versus early salvage approaches will be instrumental in refining treatment models and enhancing patient outcomes.

## Figures and Tables

**Table 1:** Characteristics of the included studies.

Study	Author, Year	Country	Sample Size	pT Stage/Risk Factors	RT Dose & Technique	Timing Post-RP	Primary Endpoint	Median Follow-Up
SWOG S8794	Thompson I et al., 2009 [38]	USA	425	pT3N0M0 (ECE, SVI, positive margins)	60–64 Gy, 2D	≤18 weeks	MFS	12.6 years
EORTC 22911	Bolla M et al., 2012 [39]	Europe	1005	pT2–3N0 with positive margins or ECE	60 Gy, 2D	≤16 weeks	Biochemical PFS	10.6 years
ARO 96-02	Wiegel T et al., 2014 [40]	Germany	307	pT3N0, undetectable PSA	60 Gy, 3D conformal	6–12 weeks	PFS	9.3 years
NCT02668718	Hackman G et al., 2019 [41]	Finland	250	pT2 with positive margins or pT3a	66.6 Gy, 3D conformal	≤12 weeks	Biochemical PFS	9.3 years

**Table 2:** Toxicity and quality of life.

Study	Author, Year	Toxicity	QoL
SWOG S8794	Thompson I et al., 2009 [38]	Not reported	Prospective QoL subset (n = 217): at 6 weeks, bowel tenderness/urgency 47% RT vs. 5% observation; urinary frequency more common with RT; no difference in ED (high in both) Global QoL: worse initially with RT, similar by year 2, then better with RT over the following 3 years
EORTC 22911	Bolla M et al., 2012 [39]	10-yr cumulative incidence: any late AE 70.8% RT vs. 59.7% (*p* = 0.001); Grade 3 late 5.3% RT vs. 2.5% (*p* = 0.052); GU ≥ G2 21.3% RT vs. 13.5% (*p* = 0.003); GI ≥ G2 2.5% vs. 1.9% (*p* = 0.47); no Grade 4 events; excess grade 1–2 largely within first 3 years	Not reported
ARO 96-02	Wiegel T et al., 2014 [40]	Late toxicity (RT arm, n = 148): 1 Grade 3 bladder; 3 Grade 2 bladder; 2 Grade 2 rectum; no Grade 4 Grade 1 late bladder/rectal: 21.9% RT vs. 3.7% (*p* < 0.0001) Urethral stricture: 3% RT vs. 1%	Not reported
NCT02668718	Hackman G et al., 2019 [41]	Any grade 3 AE: 56% RT vs. 40% (*p* = 0.016); Grade 4: 1% RT vs. 0% Most common Grade 3 AEs: ED 37% RT vs. 28%; urinary incontinence 12% vs. 5% GI disorders: Grade 1 77% RT vs. 13%; Grade 2 23% vs. 3%; Grade 3 1% vs. 1%; Grade 4 0% vs. 0% Urinary disorders: Grade 1 88% vs. 62%; Grade 2 57% vs. 38%; Grade 3 14% vs. 6%; Grade 4 0% vs. 0% Urethral stricture (Grade 3, surgery required): 12/126 (9.5%) RT vs. 3/124 (2.4%).	Patient-reported instruments: IIEF-5, IPSS, LENT-SOMA (urinary & intestinal) collected up to ~51 months Severe intestinal LENT-SOMA (grade 3–4): significantly higher with RT (modelling shows observation vs. RT OR 0.04, 95% CI 0.00–0.43; *p* = 0.008) Severe urinary LENT-SOMA: no significant difference (OR 0.76; *p* = 0.4) Severe ED by IIEF-5 over time: no significant between-group difference (OR 0.70; *p* = 0.4) Severe urinary symptoms (IPSS 20–35): trend toward more with RT (OR 0.51 for observation vs. RT; *p* = 0.061).

## Data Availability

Not applicable.

## Supplementary Materials

The following supporting information can be downloaded at https://www.mdpi.com/article/10.3390/jcm15083149/s1, Table S1: Risk of Bias using Robs 2; Table S2: GRADE certainty of evidence; Table S3. PRISMA checklist [55].
